# A Potential Prognostic Long Noncoding RNA Signature to Predict Recurrence among ER-positive Breast Cancer Patients Treated with Tamoxifen

**DOI:** 10.1038/s41598-018-21581-w

**Published:** 2018-02-16

**Authors:** Kang Wang, Jie Li, Yong-Fu Xiong, Zhen Zeng, Xiang Zhang, Hong-Yuan Li

**Affiliations:** 1 Department of the Endocrine and Breast Surgery, The First Affiliated Hospital of Chongqing Medical University, Chongqing Medical University, Chongqing, 400016 China; 2 Department of Gastrointestinal Surgery, The First Affiliated Hospital of Chongqing Medical University, Chongqing Medical University, Chongqing, 400016 China; 3 Department of the Breast and Thyroid Surgery, The Traditional Chinese Medicine Hospital, Chongqing, 400021 China

## Abstract

Limited predictable long noncoding RNA (lncRNA) signature was reported in tamoxifen resistance among estrogen receptor (ER)-positive breast cancer (BC) patients. The aim of this study was to identify and assess prognostic lncRNA signature to predict recurrence among ER-positive BC patients treated with tamoxifen. Cohorts from Gene Expression Omnibus (GEO) (n = 298) and The Cancer Genome Atlas (TCGA) (n = 160) were defined as training and validation cohort, respectively. BC relapse associated lnRNAs was identify within training cohort, and the predictable value of recurrence was assessed in both cohorts. A total of 11lncRNAs were recognized to be associated with relapse free survival (RFS) of ER-positive BC patients receiving tamoxifen, who were divided into low-risk and high-risk group on basis of relapse risk scores (RRS). Multivariate cox regression analyses revealed that the RRS is an independent prognostic biomarker in the prediction of ER-positive BC patients’ survival. GSEA indicated that high-risk group was associated with several signaling pathways in processing of BC recurrence and metastasis such as PI3K-Akt and Wnt signaling. Our 11-lncRNA based classifier is a reliable prognostic and predictive tool for disease relapse in BC patients receiving tamoxifen.

## Introduction

As a formidable health problem for women around the world^1^, breast cancer (BC) is the most frequently diagnosed cancer and the arch-criminal of cancer death among women. In the United States, estimated 252,710 new cases will occur, and 40,610 of them will die in 2017, accounting for 30% of new cases and 14% of deaths of all sites cancers^2^. BC can be basically divided into four different subtypes including luminal A and luminal B accounting for approximately 70% of all BCs and marked with estrogen receptor (ER)-positive^3,4^, human epidermal growth factor receptor 2 (HER-2) overexpression and triple negative with poorer outcomes^5,6^. Disparity of therapy strategies according to pathological characteristics has been well established.

Tamoxifen is a selective ERα antagonist, the drug of first-line endocrine treatment, which is still widely prescribed in pre-menopausal women Luminal BC patients. Although tamoxifen can reduce the risk of BC recurrence by 41% and mortality by 34% after 5 years’ treatment^7^, almost 30% of ongoing treated with tamoxifen may develop resistance to the drug, which leads to cancer recurrence or metastasis with poor disease-free or overall survival^8^. Some biomarkers were proposed to predict the drug resistance of these ER-positive patients primarily treated with tamoxifen, such as ER expression, progesterone receptor (PR) expression, HER2 and multigene signatures^9^. Additionally, age^10,11^, American Joint Committee on Cancer (AJCC) stage^12^ and lymph nodes ratio (LNR)^13,14^ that is the ratio of the numbers of metastatic lymph nodes to those of the dissected lymph nodes were also important prognostic factors on prediction of BC recurrence, which were also considered as potential confounding factors when developing new biomarkers.

Along with the exploration going to a deeper and finer status, the role of long noncoding RNAs (lncRNAs) in the formation of tamoxifen resistance attracts much more attention^15–17^. LncRNAs are a class of RNA molecules more than 200 nucleotides in length and without protein-coding capacity^18,19^. They participates in all levels of nuclear architecture and gene expression, involving in telomere stability, long range DNA looping and trans-chromosomal interactions, X-chromosome inactivation, imprinting, formation of nuclear sub-compartments, fine regulation of epigenetic modification, regulation of RNA splicing and maturation, control of mRNA and protein stability^20–22^. It has been demonstrated that the dysregulation of lncRNA is associated with various kinds of human diseases, such as cancers, diabetes, chronic hepatitis C, ankylosing spondylitis, autism spectrum disorder^23–27^. Its predicted value for prognosis has been confirmed in several cancers including lung cancer, gallbladder cancer, ovarian cancer, gastric cancer and colorectal cancer^28–33^. Accordingly, previous studies had identified that overexpression of LncRNA BCAR4 in tamoxifen-sensitive ZR-75-1 cells blocked the anti-proliferative effects of tamoxifen, increasing the tamoxifen resistance^34,35^. Nevertheless, limited number of LncRNAs were proposed as clinically relevant biomarkers for tamoxifen resistance, and searching a lncRNA signature might be concrete predictive and prognostic value in the management of ER-positive BC receiving tamoxifen.

In this study, we used data acquired from The Cancer Genome Atlas (TCGA, http://cancergenome.nih.gov/) and Gene Expression Omnibus (GEO, https://www.ncbi.nlm.nih.gov/gds) whose gene expression microarray data can be mined to develop LncRNAs profiling^36^ to identify the predicted effects of lncRNAs on the relapse of ER-positive BC patients treated with tamoxifen.

## Materials and Methods

### ER-Positive BC treated with tamoxifen Datasets

ER-positive BC datasets corresponding clinical data were downloaded from the GEO and TCGA as training and external validation series, respectively. Only the ER-positive BC patients who were primarily treated with tamoxifen as the unique endocrine therapy other than mixed endocrine therapy such as alternative aromatase inhibitors (AIs) were included. The endpoint is defined relapse free survival (RFS), which is an interval from time of BC diagnosed to disease relapse or metastasis. GSE17705 dataset was screened, and 298 eligible patients treated with tamoxifen for 5 years with clinicopathological and survival data were included in our study as training cohort. After removing corresponding BC patients from TCGA database without clinicopathological data such as age, AJCC stage, LNR, chemotherapy and relapse or progress status, 160 ER-positive BC patients were enrolled finally, which were applied for validation cohort and nomogram construction. Eligible samples were identified from GSE17705 (n = 298) on basis of Affymetrix HG-U133A platform and TCGA dataset (n = 160), whose clinicopathological characteristics in two datasets are presented in Table 1.

**Table 1:** Clinical Characteristics of the Patients.

Variable	GSE17705 cohort	TCGA cohort
**No. of patients**	298	160
**Age, year, mean (SD)**	64(10)	53(13)
**T stage, No. (%)**		
I	133(45)	37(23)
II	151(51)	102(64)
III	23(8)	21(13)
NR	1(1)	0(0)
**Lymph node status, No. (%)**		
Positive	118(40)	75(47)
Negative	180(60)	58(36)
NR	0(0)	27(17)
**AJCC stage, No. (%)**		
I	88(30)	25(16)
II	183(61)	95(59)
III	22(7)	36(23)
NR	5(2)	4(2)
**PR status, No. (%)**		
Positive	77(26)	124(78)
Negative	22(7)	19(12)
NR	199(67)	17(10)

Abbreviation: TCGA, The Cancer Genome Atlas; SD, standard deviation; NR, not recorded; AJCC, American Joint Committee on Cancer; PR, progesterone receptor.

### LncRNA Expression Data Processing and Profile Mining

Raw microarray data files of GSE17705 was retrieved from GEO database, and we adjusted the background using the Robust Multichip Average (RMA) algorithm actualized by R package ‘affy’^37^. LncRNA profile mining was mainly referenced to method proposed by Du *et al.*^38^. Briefly, we mapped the probe sets IDs from Affymetrix HG-U133A to human genome, and SeqMap^39^ was used to avoid mismatching. Then, the chromosomal positions according these probe IDs were used to identify corresponding positions of LncRNA. Finally, 569 annotated lncRNA transcripts with 742 corresponding Affymetrix probe IDs were generated. The expression profiling of multiple probe IDs mapped to unique LncRNA was calculated as arithmetic mean of the values. Similarly, we downloaded expression information from TCGA BC RNA-sequencing database of LncRNA using The Atlas of Noncoding RNAs Cancer (TANRIC)^40^, and then annotated each included sample according to barcode ID.

### Statistical

To identify LncRNAs predictive of a RFS, a univariate Cox proportional hazards regression analysis was performed to assess relationship between expression of LncRNAs level and RFS among training series, which was considered statistically significant if their P values were less than or equal to 0.01. Then we conducted stepwise multivariate Cox hazard regression analysis using above LncRNAs to yield a set of genes ultimately that had independent effects on RFS, and a relapse risk score (RRS) for predicting RFS was calculated by taking into account the expression of gene and correlation coefficient as following:

1 $$RRS=\sum _{i=1}^{n}({\rm{E}}({\rm{i}})\ast C(i))$$

where n is the number of selected LncRNAs, E is the expression level of LncRNAs and C is the coefficient generated from multivariate Cox regression. Considering the practice of this prognostic model, we assigned each patient a RRS combined linearly by expression of significant LncRNAs and their respective coefficients. Then, we divided training and validation cohort into low-risk group and high-risk group according to RRS, respectively, and the third quantile RRS is defined as the cut-off value depended on receiver operating characteristic (ROC). RFS curve was estimated between high-risk group and low-risk group using Kaplan Meier method, and log-rank tests were used to evaluate the differences between them. ROC curve was conducted to assess sensitivity and specificity of this RRS on basis of selected LncRNAs. To further analysis the independent association between RRS and RFS, univariate and multivariate Cox proportional hazards regressions were applied using clinicopathological factors and RRS. This action was executed only within TCGA cohort, which had relatively comprehensive clinical data. Additionally, a nomogram including factors mentioned before was constructed, and validated internally through 200 bootstrap resamples. Concordance index (C-index) was calculated for the evaluation of the performance of predicting and discrimination ability by test concordance between predicted probability and actual outcome. Similarly, to achieve visualization, calibration of this nomogram was conducted by comparing the predicted survival with the observed survival in both, and a 45-degree diagonal line was deemed to a perfectly calibrated model.

### Gene Set Enrichment Analysis

To reversely validate the functional prediction of identified lncRNAs set, we conducted gene set enrichment analysis (GSEA) based on GSE17705 cohort. GSEA was performed by the JAVA program (http://www.broadinstitute.org/gsea) through MSigDB C2 CP: Canonical pathways gene set collection (1320 gene sets available). Gene sets with a false discovery rate (FDR) value < 0.05 after performing 1,000 permutations were considered to be significantly enriched^41^, and selected recurrence or metastasis related enrichment results were visualized by Cytoscape (version 2.8.2) and the Enrichment Map software^42^.

All P or adjusted P values reported were two-sided, which less than 0.05 were considered statistically significant. All of statistical tests were done with SPSS 23 (SPSS, Chicago, IL, USA), R software (version 3.2.5) with Bioconductor^43^ and rms packages.

## Results

### Identification of LncRNA genes associated with relapse in the training cohort

**Figure 1:**
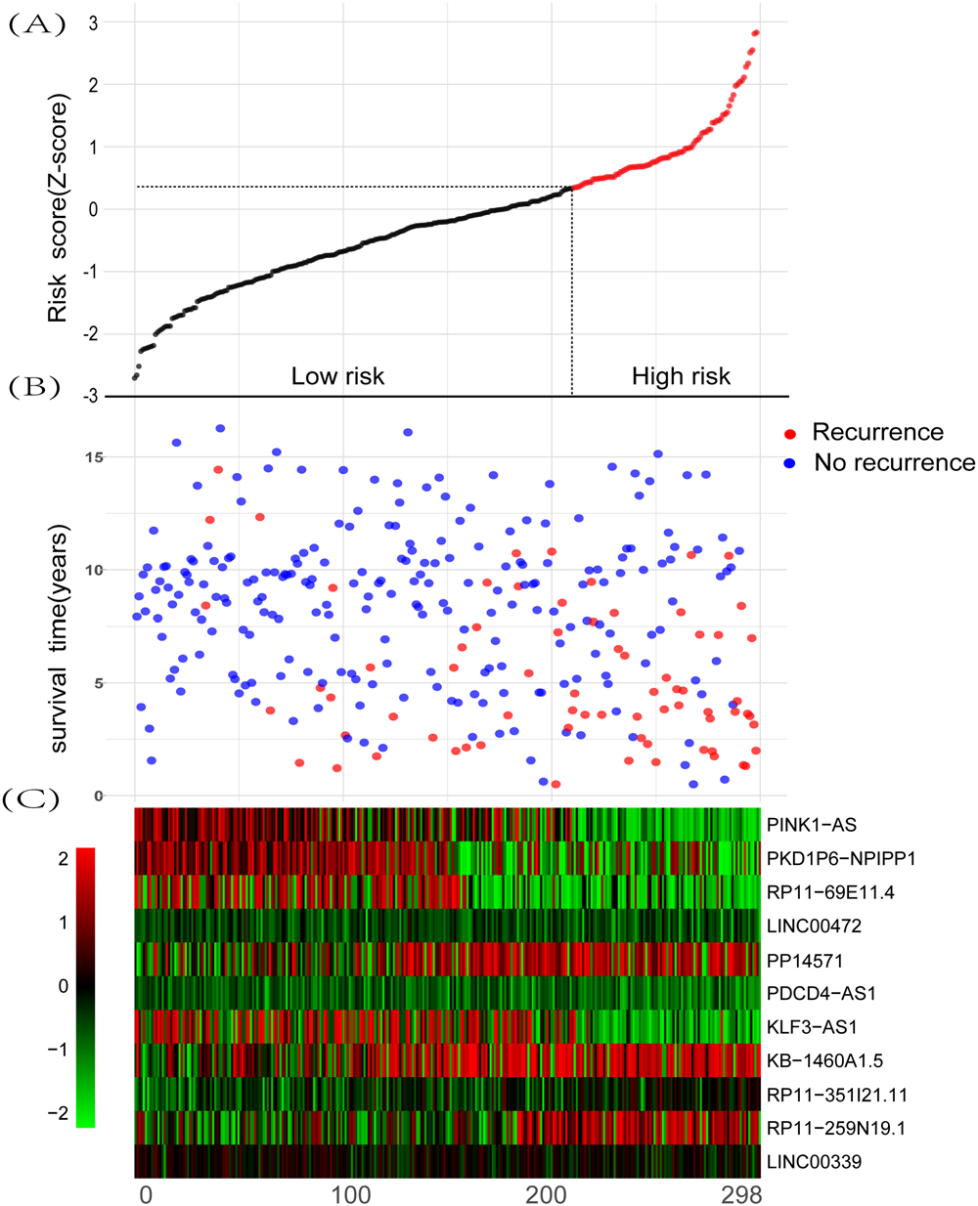
11-LncRNA signature biomarker characteristics in the GSE17705 cohort (N = 298). (**A**) 11-lncRNA expression and risk score distribution by z-score. The risk scores for all patients in GSE17705 cohort are plotted in ascending order and marked as low risk (black) or high risk (red), as divided by the threshold (vertical black line). (**B**) Patients’ recurrence status and time; (**C**) heatmap of the lncRNA expression profiles. Rows represent lncRNAs, and columns represent patients. Red indicated higher expression and black indicated lower expression.

**Figure 2:**
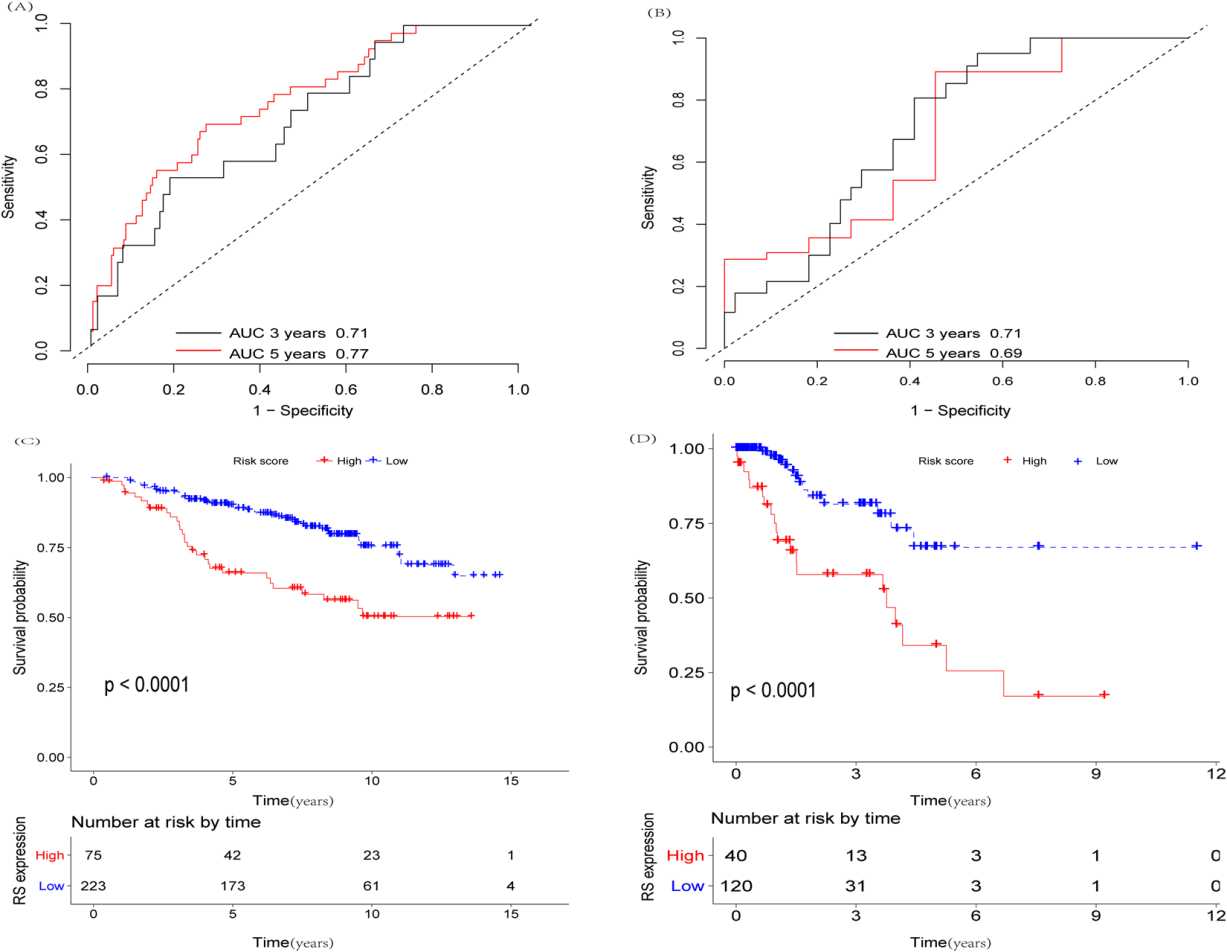
ROC cures and Kaplan-Meier estimates of 3-year and 5-year RFS. ROC curves showed sensitivity and specificity of 3-year and 5-year RFS prediction by the 11-lncRNA risk score within (**A**) GSE17705 cohort (n = 298) and (**B**) TCGA cohort (n = 160). Kaplan-Meier plots were used to visualize the survival probabilities for the low-risk versus high-risk group of patients determined on the basis of the third quartile risk score from the training-set patients within (**C**) GSE17705 cohort (n = 298) and (**D**) TCGA cohort (n = 160) (all P < 0.0001).

GES17705 (n  =  298) was used to explore prognostic LncRNAs, which was larger database in this study. A univariate Cox proportional hazards regression analysis was performed to identify whether each LncRNA gene expression level was associated with RFS, and we identified a set of 23 genes (PINK1.AS, RP11.932O9.9, RP11.259N19.1, RP3.339A18.6, KLF3.AS1, LINC00339, LINC00472, RP11.351I21.11, RP11.72I8.1, KB.1460A1.5, RP1.223E5.4, PKD1P6.NPIPP1, RP11.499P20.2, PDCD4.AS1, RP6.74O6.2, KB.1592A4.15, PP14571, RP11.69E11.4, MGC16275, RP11.105C19.2, AC093110.3, LLNLR.284B4.1, RP11.488C13.7) were significantly correlated with RFS (p < 0.05, Supplementary Table S1). Stepwise multivariate Cox proportional hazards regression analysis was conducted to select final 11 included LncRNAs, including PINK1.AS, RP11.259N19.1, KLF3.AS1, LINC00339, LINC00472, RP11.351I21.11, KB.1460A1.5, PKD1P6.NPIPP1, PDCD4.AS1, KLF3.AS1 PP14571, RP11.69E11.4. Among those gens, the negative coefficients for the eight genes (PINK1.AS, KLF3.AS1, LINC00339, LINC00472, RP11.351I21.11, PKD1P6.NPIPP1, PDCD4.AS1, RP11.69E11.4) indicated high level of expression were associated with longer RFS. The positive coefficients for remaining 3 genes (RP11.259N19.1, KB.1460A1.5, PP14571) revealed that high expression of them was associated with shorter RFS. Accordingly, a RRS was established to predict the relapse risks of BC patients treated with tamoxifen, as following: RRS = (−0.67223* expression value of PINK1.AS) + (0.38401* expression value of RP11.259N19.1) + (−0.18630* expression value of KLF3.AS1) + (−0.19157* expression value of LINC00339) + (−0.21613*expression value of LINC00472) + (−0.20857*expression value of RP11.351I21.11) + (0.22666*expression value of KB.1460A1.5) + (−0.50148* expression value of PKD1P6.NPIPP1) + (−0.23588* expression value of PDCD4.AS1) + (0.16569* expression value of PP14571) + (−0.23448* expression value of RP11.69E11.4). Distribution of the eleven-lncRNA risk score, relapse status, and LncRNAs expression of training patients were shown in Fig. 1. After conducting ROC analysis, the prognostic power of 3-year or 5-year RFS predicted by eleven-LncRNAs risk score was evaluated within GSE17705. As shown in Fig. 2A, the sensitivity, specificity of RRS were 0.67 and 0.74, respectively, and the area under curves (AUCs) of RRS for prediction 3-year and 5-year RFS were 0.71 and 0.77, respectively, indicating that the performance of eleven-LncRNAs based RRS was mediocre. The training cohort was divided into high-risk (n = 74) and low-risk group (n = 224) according to the third quartile risk score as the cut-off value. Kaplan-Meier analysis revealed that patients in high-risk group had significantly shorter median DFS than those in low-risk group (log-rank test P < 0.0001) (Fig. 2C).

### The 11-lncRNA signature and the patients’ survival in the external validation cohort

**Figure 3:**
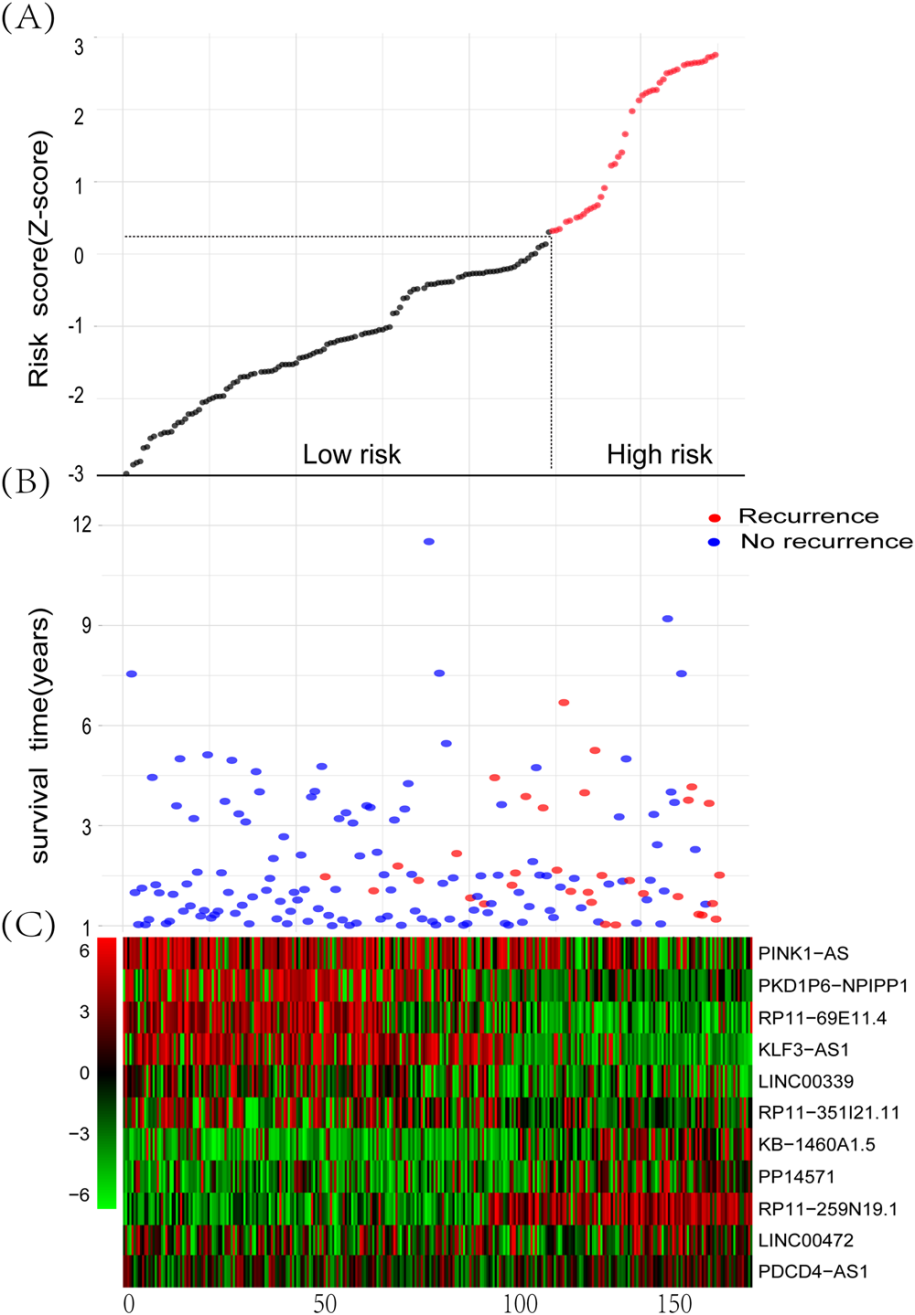
11-LncRNA signature biomarker characteristics in the TCGA cohort (N = 160). (**A**) 11-lncRNA expression and risk score distribution by z-score. The risk scores for all patients in TCGA cohort are plotted in ascending order and marked as low risk (black) or high risk (red), as divided by the threshold (vertical black line). (**B**) Patients’ recurrence status and time; (**C**) heatmap of the lncRNA expression profiles. Rows represent lncRNAs, and columns represent patients. Red indicated higher expression and black indicated lower expression.

To validate the prognostic power of the eleven-lncRNA signature for the prediction of relapse risk, the TCGA cohort (n = 160) use the same formula to classify patients into high-risk group (n = 40) and low-risk group (n = 120) using the third quartile RRS of validation series as the cut-off point. ROC suggested that AUC of 0.71 and 0.69 revealed general predictive performances of 3-year and 5-year RFS, respectively (Fig. 2B). In the consistence with the findings described above, patients in the high-risk group had significantly shorter median RFS than those in the low-risk group (log-rank test P < 0.0001) (Fig. 2D). The distribution of the eleven-lncRNAs risk score, the relapse status of the BC patients and the lncRNA expression signature were also obtained (Fig. 3).

### Independence of RFS prediction by the 11-lncRNA signature from other clinicopathological variables

**Figure 4:**
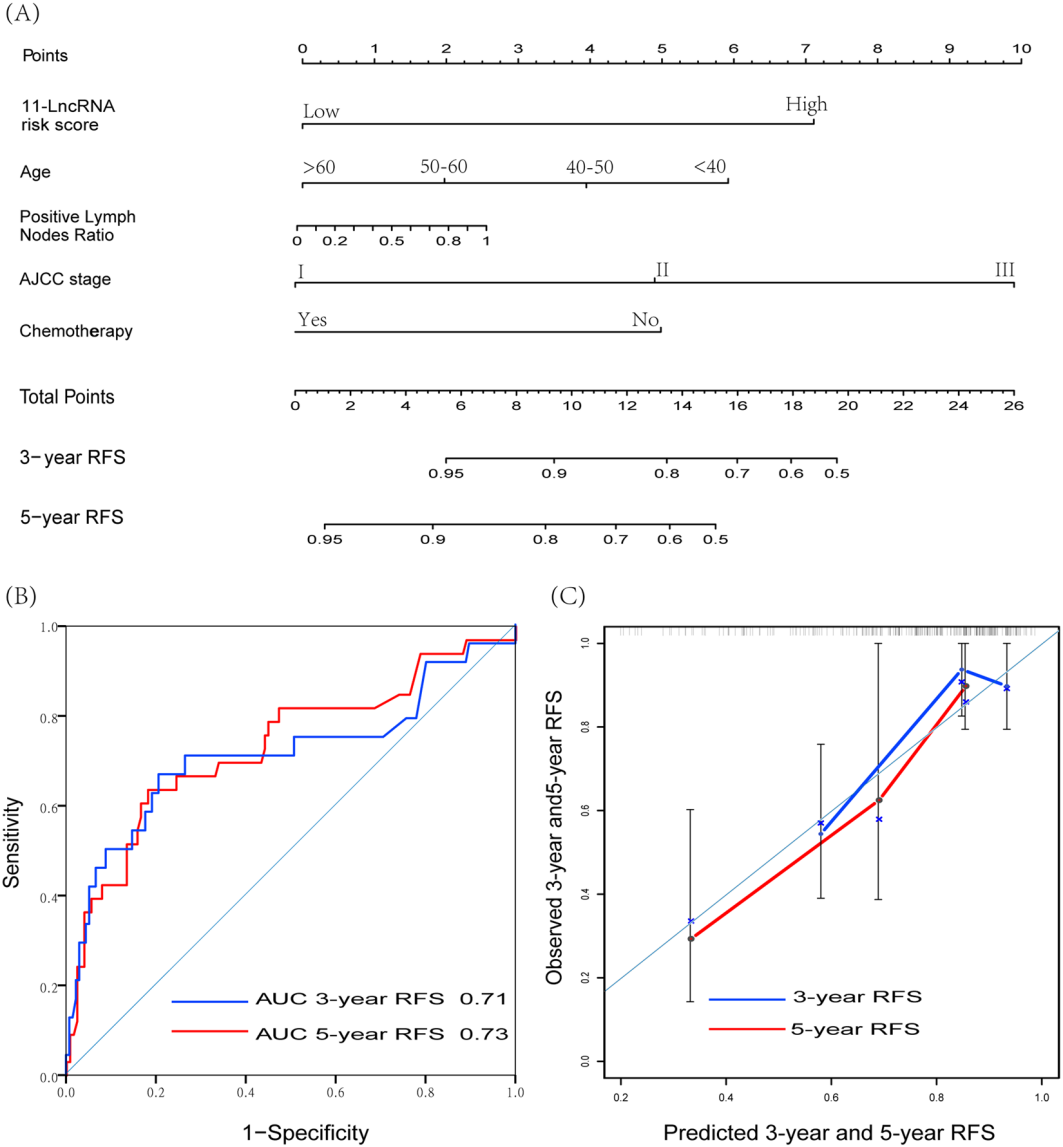
Nomogram, ROC curves and the internal calibration for predicting 3-year and 5-year RFS within TCGA cohort (n = 160). The nomogram was built using 11-lncRNA risk score, age, positive lymph nodes ratio, AJCC stage, and chemotherapy. (**A**) Nomogram for predicting proportion of patients with 3-year and 5-year RFS. (**B**) ROC curve showed sensitivity and specificity of the nomogram. (**C**) Plots depict the calibration of model in terms of agreements between predicted and observed 3-year and 5-year RFS. Model performance is shown by the plot, relative to the 45-degree line, which represents perfect prediction.

To further test whether the 11-lncRNA signature was independent of other clinicopathological variables, we conducted multivariate Cox regression analysis using RRS, age, AJCC stage, chemotherapy status and LNR as covariables in the TCGA cohort (n = 160). The results suggested that the 11-lncRNA signature risk score (RRS) maintained a significant correlation with RFS after adjustment for other clinical variables (HR = 3.56, 95%CI = 1.46–8.66) (Table 2), indicating that RRS is an independent relapse predictive factor. Additionally, a nomogram was constructed using above variables, which was shown as Fig. 4A. Corresponding ROC analyses were conducted according to 3-year and 5-year RFS (Fig. 4B), and yielded AUC of 0.71 and 0.73 indicated that RRS combined clinicopathological variables can generally predict 3-year and 5-year RFS, respectively. In internal validation, C-indexes for the nomograms to predict RFS were 0.78 (95% CI, 95%CI = 0.67 to 0.89), whose calibration plots for prediction of 3-year and 5-year RFS were presented in Fig. 4C.

**Table 2:** Univariate and Multivariable Cox Regression Analysis in TCGA Cohort.

Variable	Relapse Free Survival			
	Univariate Analysis		Multivariate Analysis	
	HR (95% CI)	P value	HR (95% CI)	P value
**11-LncRNA risk score (n = 160)**				
Low	Reference		Reference	
High	3.30(1.66–6.57)	0.001	3.56(1.46–8.66)	0.005
**Age (year)**				
<40	Reference		Reference	
40–50	0.64(0.24–1.69)	0.37	0.44(0.15–1.34)	0.15
50–60	0.42(0.12–1.44)	0.17	0.35(0.09–1.31)	0.12
>60	1.19(0.46–3.04)	0.72	0.36(0.10–1.36)	0.13
**AJCC stage**				
I	Reference		Reference	
II	1.97(0.57–6.82)	0.29	4.06(0.82–29.09)	0.09
III	4.89(1.39–17.15)	0.01	6.60(1.17–37.34)	0.03
LNR*	3.60(0.97–13.43)	0.06	2.28(0.28–18.76)	0.45
**Chemotherapy**				
Yes	Reference		Reference	
No	0.64(0.31–1.32)	0.23	0.41(0.14–1.22)	0.11

*In Cox regression analysis, LNR was considered as continuous variable.

Abbreviations: HR, hazard ratio; CI, confidence interval; LNR, positive lymph node ratio.

### Functional prediction of the 11-lncRNA signature

**Figure 5:**
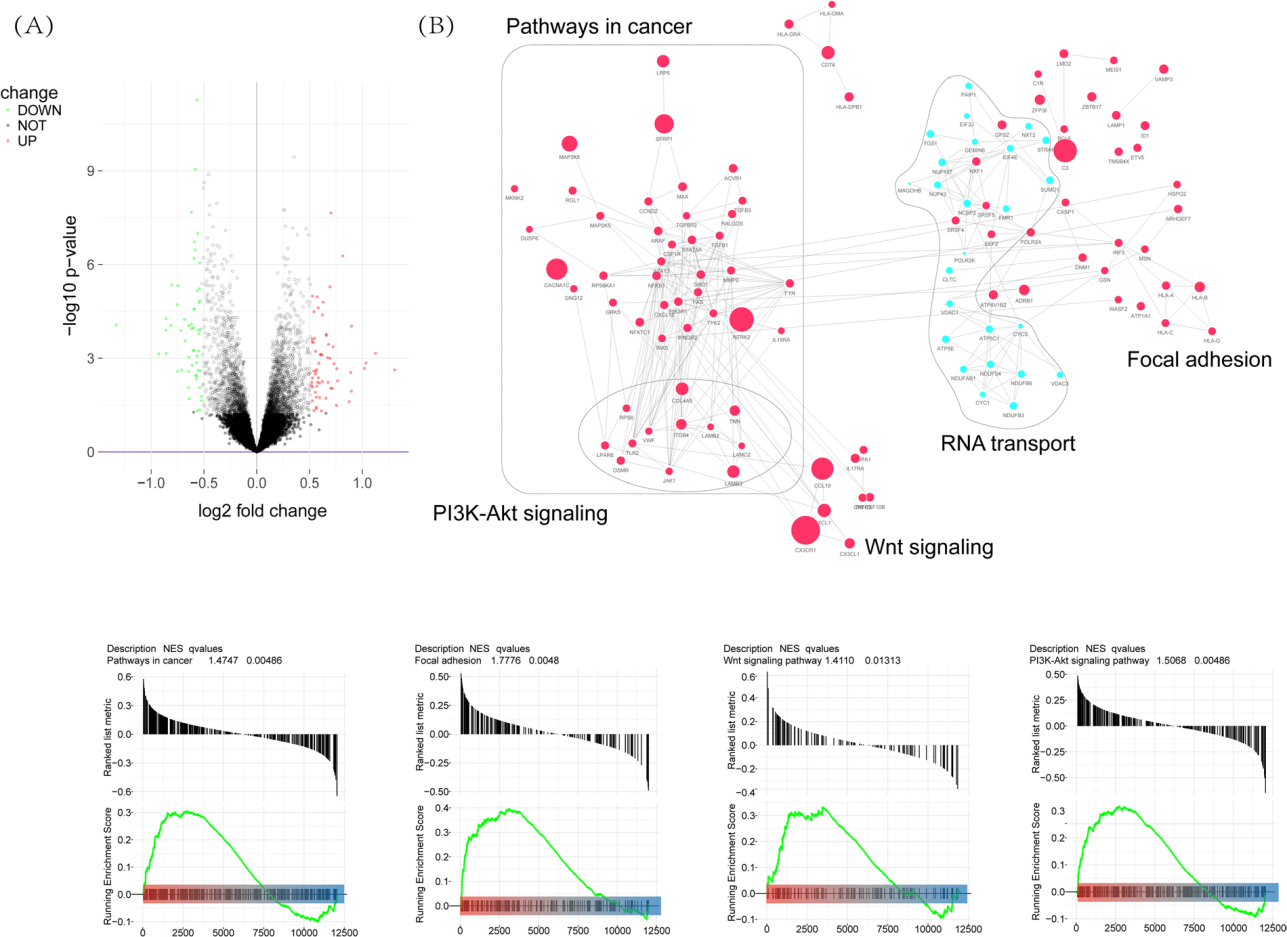
(**A**) Gene Set Enrichment Analysis Delineates Biological Pathways and Processes associated with risk score within GSE17705 cohort. (A) Volcano Plot with t-statistics. Circles show potentially differential genes between low-risk and high-risk group based on 11-lncRNA risk scores. The green and red circle were up and down genes, respectively. (**B**) Cytoscape and Enrichment Map were used for visualization of the GSEA results. Nodes represent enriched gene sets, which are grouped and annotated by their similarity according to related gene sets. Enrichment results were mapped as a network of gene sets (nodes). Node size is proportional to the total number of genes within each gene set. Proportion of shared genes between gene sets is represented as the thickness of the green line between nodes.

To infer the potential function of the 11-LncRNAs signature in prediction of BC relapse, we performed GSEA to identify associated biological processes and signaling pathways based on GSE17705 datasets. The gene sets from high-risk group compared with low-risk defined before was employed to select significant individuals (adjusted P < 0.05) (Fig. 5A) (Supplementary Table S2), and then relapse or metastasis related sets of them were visualized as interaction networks with Cytoscape and Enrichment Map (Fig. 5B). Several relapse or metastasis-related pathways like PI3K-Akt signaling, Wnt signaling and focal adhesion were found from up-regulation gene sets (Fig. 5B).

## Discussion

In this study, we developed and validated a novel prognostic tool based on 11-lncRNAs to improve the prediction of disease recurrence for ER-positive BC patients who were primarily treated with tamoxifen as unique endocrine therapy. Our results showed that this tool can successfully classify patients into high-risk and low-risk groups, and differences in 3-year/5-year RFS were identified in training and validation cohorts. In addition, the 11-lncRNAs signature was proved to be independent from other clinicopathological variables, and GSEA was conducted on basis of RRS to validate the functional differences. A comprehensive and practical nomogram was construct to predict the individualized recurrence, which provided opportunities for clinicians to classify the patients according to these valid tools.

LncRNA LINC00472 is located on chromosome 6q13, and the verified transcript has 2,933 bp. Previous *in vitro* study with meta-analysis suggested that LINC00472 is a tumor suppressor in breast cancer, whose potential prognostic and predictive value should be evaluated^44^. Similarly, Shen *et al*. indicated that LINC00472 expression in breast tumors predicted the recurrence of grade II breast cancer^45^. Interestingly, high expression of LINC00472 was often more frequently in low grade and early stage epithelial ovarian cancer compared to high grade tumors and late stage^46^. Moreover, integrated analysis of LncRNA-associated competing endogenous RNAs (ceRNAs) network revealed that LINC00472 was positively correlated with OS of lung adenocarcinoma, and the correlation between LINC00472 and AFAP1-AS1, one of the most common biomarkers to predict the outcomes of cancer patients, was also confirmed^47,48^. LncRNA KB.1460A1.5 was only proposed in Alzheimer’s disease, suggesting this lncRNA with high topological features in the intracellular neurofibrillary tangles may play an important role in Alzheimer’s disease. Unexpectedly, related reports were not found in field of oncology. Among the 11 LncRNAs, except for those mentioned above, other lncRNAs are either poorly investigated or have not been reported, which means our findings suggested further research for them is imperative.

With the development of next generation sequencing technologies and the rise of transcriptomics, a class of noncoding RNA attracts people’s attention such as MicroRNAs (miRNAs) and lncRNAs. Several miRNAs are thought to involve in tamoxifen resistance by directly inhibiting the expression of ER-α like miR-221/222^49^ and mi-342^50^, or mediately regulating genes related to cell survival and metastasis, such as miR-451^51^, miR-375^52^, miR-15a/16^53^, miR-200s^54^. Zhang H *et al*. reported that downregulation of lncRNA-ROR could enhance the sensibility of breast cancer cells to tamoxifen by increasing miR-205 expression^15^. LncRNA UCA1 could enhance tamoxifen resistance via inhibiting mTOR signaling pathway, activating wnt/β-catenin signaling pathway, or through a miR-18a-HIF1α regulatory feedback loop^16,55,56^. LncRNA HOTAIR could promote the ligand-independent ER activities and thereby contribute to tamoxifen resistance^57^.

Accumulating evidences indicate that the altered expression of ERα, crosstalk with other signaling like HER-2, PI3K and MAPK signaling, mutation of ESR1 and decreased metabolism of the drug may contribute to the acquiring resistance of tamoxifen^8,58–60^, of which the phosphatidylinositol 3-kinase (PI3K)/Akt/mammalian target of rapamycin (mTOR) signaling pathway was identified within results of GSEA in this study. PI3K/Akt and mTOR are two important signaling pathways controlling series of biological processes like cell growth and proliferation, apoptosis, cell-cycle, DNA repair, angiogenesis and glucose metabolism^61^. Ample preclinical studies have indicated that PI3K/Akt/mTOR signaling pathway is co-related with endocrine resistance. The crosstalk between ER and PI3K/Akt/mTOR signaling pathway is a key mechanism by which tamoxifen resistance happens. Yamnik, R. L. *et al*. found that S6 kinase 1, a substrate of mTOR complex 1 (mTORC1) could phosphorylate the function domain 1 of ER, which leaded to an estrogen-independent activation of the ER-α, thereby resulting in tamoxifen resistance^62^. Otherwise, clinical data showed that the activation of Akt or reduction of PTEN, a tumor suppressor mediating dephosphorylation of PIP3 to PIP2, were associated with tamoxifen resistance in both metastatic and recurrent breast cancer patients^63,64^. Everolimus, a mTOR inhibitors, has been approved by FDA for the treatment of hormone receptors positive and HER2 negative advanced postmenopausal breast cancer patients, hoping to overcome or slowdown the development of endocrine resistance.

So far, a serious of studies have demonstrated that the wnt/β-catenin signaling pathway is co-related with embryogenesis, self-renewal of adult tissues and tumorigenesis via controlling cell cell morphology, differentiation, proliferation, migration, apoptosis, and even stem cell self-renewal^65–71^. And the accumulation of β-catenin in the nucleus or cytoplasm has been found in approximately 50% of breast cancers, which predicted a poor prognosis^72^. Meanwhile, wnt/β-catenin has the potential to promote cancer malignant development through motivating the EMT process to facilitate cancer metastasis or inducing therapeutic resistance^73,74^. Interestingly, lncRNA HNF1A-AS1, lncRNA CCND2-AS1, lncRNA CRNDE, lncRNA CASC2 were revealed that promotional effects on cell proliferation via activation of Wnt/β-catenin cascade in osteosarcoma, glioma, colorectal cancer and bladder cancer were documented, respectively^75–78^. Additionally, lncRNA UCA1 promotes epithelial-mesenchymal transition of BC cells via enhancing Wnt/β-catenin signaling pathway to facilitate its metastasis^79^, and better reactions to tamoxifen through inhibiting Wnt/β-catenin pathway were found when knock-downing of lncRNA UCA1 could^56^.

Some limitations of our study should be acknowledged. Firstly, GSE17705 data sets included in this study was profiled through Affymetrix Human Genome U133A Array, and only part of possible lncRNAs were mined. The incomplete candidate lncRNAs maybe cannot represent the whole biological function of this noncoding RNA, and more large-scale studies were urgently needed to validate reliability of our model. Secondly, eligible participants from two cohorts all received tamoxifen as primarily endocrine therapy, but status and duration of this prescription was poorly reported. The GSE17705 cohort involved 298 BC patients treated with 5-year tamoxifen, whereas TCGA cohort was identified by therapy data, which is one of possible reasons of imperfect validation. Lastly, no experimental validation was acquired to reveal the underlying mechanisms of 11-lncRNAs based prognostic model, and further experimental studies were needed to supply the better understanding of this 11-lncRNAs signature.

In conclusion, our findings showed that the 11-lncRNA based classifier is a reliable prognostic and predictive tool for disease relapse in BC patients receiving tamoxifen, which is independent from traditional clinicopathological factors. Developed nomograms is also available to predict whether patients can benefit from tamoxifen therapy, which are valid tools to illustrate the visualized results.

## Electronic supplementary material

Supplementary Table S1

Supplementary Table S1
