# Supplementary material for: A Potential Prognostic Long Noncoding RNA Signature to Predict Recurrence among ER-positive Breast Cancer Patients Treated with Tamoxifen
Source: Sci Rep. 2018 Feb 16;8:3179. doi: 10.1038/s41598-018-21581-w (PMC5816619; doi:10.1038/s41598-018-21581-w)
Supplement: Supplementary file 1 — Supplementary Table S1 [file 41598_2018_21581_MOESM1_ESM.pdf]

A Potential Prognostic Long Noncoding RNA Signature to Predict Recurrence among ER-positive Breast Cancer Patients Treated with Tamoxifen

Kang Wang, Jie Li, Yong-Fu Xiong, Zhen Zeng, Xiang Zhang, Hong-Yuan Li

Supplementary Table S1 A univariate Cox proportional regression analysis identified LncRNA genes expression level associated with RFS in GSE17705 ($p < 0.05$)

Genes	Ensembl ID	Chromosomal location	coef	exp(coef)	se(coef)	z	Pr(>|z|)	exp(coef)	exp(-coef)	lower .95	upper .95
PDCD4-AS1	ENSG00000203497	10q25.2	-0.00436	0.995651	0.001462	-2.98036	0.002879	0.995651	1.004368	0.992801	0.998509
RP1-223E5.4	ENSG00000261071	6p23	0.00406	1.004068	0.001374	2.955127	0.003125	1.004068	0.995948	1.001368	1.006776
KB-1592A4.15	ENSG00000197210	22q11.21	-0.00407	0.995941	0.001411	-2.88336	0.003935	0.995941	1.004075	0.993191	0.998698
LINC00472	ENSG00000233237	6q13	-0.00409	0.995915	0.001432	-2.85817	0.004261	0.995915	1.004102	0.993123	0.998714
RP11-72I8.1	ENSG00000260735	16p13.11	-0.00407	0.99594	0.001437	-2.83089	0.004642	0.99594	1.004077	0.993138	0.998749
AC093110.3	ENSG00000238018	2p16.2	-0.00408	0.99593	0.001442	-2.82893	0.00467	0.99593	1.004086	0.99312	0.998748
RP11-259N19.1	ENSG00000272711	2p12	0.003713	1.00372	0.001418	2.618917	0.008821	1.00372	0.996294	1.000935	1.006513
KLF3-AS1	ENSG00000231160	4p14	-0.0036	0.99641	0.001416	-2.54033	0.011075	0.99641	1.003603	0.993649	0.999179
RP11-488C13.7	ENSG00000258610	14q24.3	-0.00356	0.996447	0.001433	-2.48361	0.013006	0.996447	1.003566	0.993652	0.99925
RP6-74O6.2	ENSG00000215859	1q21.2	-0.00343	0.996579	0.001404	-2.43995	0.014689	0.996579	1.003433	0.99384	0.999326
RP11-105C19.2	ENSG00000260973	16p12.2	-0.00342	0.996587	0.001434	-2.38409	0.017122	0.996587	1.003424	0.99379	0.999392
RP3-339A18.6	ENSG00000233250	Xp11.22	0.003343	1.003349	0.001407	2.376092	0.017497	1.003349	0.996662	1.000586	1.00612
RP11-69E11.4	ENSG00000182109	1p34.3	-0.0034	0.996608	0.001447	-2.34892	0.018828	0.996608	1.003404	0.993786	0.999437
KB-1460A1.5	ENSG00000261087	8q22.3	0.003242	1.003248	0.001412	2.296777	0.021632	1.003248	0.996763	1.000476	1.006028
PKD1P6-NPIPP1	ENSG00000270580	16p13.11	-0.00332	0.99669	0.001474	-2.24956	0.024477	0.99669	1.003321	0.993814	0.999573
RP11-932O9.9	ENSG00000269930	15q13.2	-0.00308	0.996926	0.0014	-2.19843	0.027919	0.996926	1.003083	0.994193	0.999666
PP14571	ENSG00000218416	2q37.3	0.003032	1.003037	0.001391	2.179243	0.029314	1.003037	0.996972	1.000305	1.005776
RP11-499P20.2	ENSG00000240291	10p12.31	-0.00297	0.997036	0.001399	-2.12179	0.033855	0.997036	1.002973	0.994306	0.999774
MGC16275	ENSG00000246731	17q25.1	-0.00304	0.996969	0.00144	-2.10799	0.035032	0.996969	1.00304	0.994159	0.999787
PINK1-AS	ENSG00000117242	1p36.12	-0.00296	0.997043	0.001426	-2.07708	0.037794	0.997043	1.002966	0.99426	0.999833
LINC00339	ENSG00000218510	1p36.12	-0.00277	0.997229	0.001367	-2.0299	0.042367	0.997229	1.002778	0.994561	0.999904
RP11-351I21.11	ENSG00000270074	8p23.1	-0.00285	0.997157	0.00141	-2.01967	0.043417	0.997157	1.002851	0.994406	0.999916
LLNLR-284B4.1	ENSG00000274177	19p13.3	-0.00276	0.997248	0.001372	-2.00857	0.044583	0.997248	1.002759	0.994571	0.999933
