# Supplementary material for: A Potential Prognostic Long Noncoding RNA Signature to Predict Recurrence among ER-positive Breast Cancer Patients Treated with Tamoxifen
Source: Sci Rep. 2018 Feb 16;8:3179. doi: 10.1038/s41598-018-21581-w (PMC5816619; doi:10.1038/s41598-018-21581-w)
Supplement: Supplementary file 2 — Supplementary Table S1 [file 41598_2018_21581_MOESM2_ESM.pdf]

A Potential Prognostic Long Noncoding RNA Signature to Predict Recurrence among ER-positive Breast Cancer Patients Treated with Tamoxifen

Kang Wang, Jie Li, Yong-Fu Xiong, Zhen Zeng, Xiang Zhang, Hong-Yuan Li

Supplementary Table S2 LncRNA signature related signaling pathways with positive enrichment score ranked by normalizing enrichment scores.

ID (GSE17705)	Description	setSize	Enrichment Score	(Normalizing Enrichment Scores) NES	pvalue	p.adjust	qvalues	RANK AT MAX	LEADING EDGE
hsa05168	Herpes simplex	157	0.461990135	2.035402077	0.001718	0.013287	0.004865	1532	tags=27%, list=13%, signal=24%
hsa04060	Cytokine-cytokine receptor interaction	214	0.443225982	2.031419397	0.001704	0.013287	0.004865	1604	tags=29%, list=13%, signal=26%
hsa04510	Focal adhesion	181	0.396553821	1.777673644	0.001727	0.013287	0.004865	3098	tags=43%, list=26%, signal=32%
hsa05224	Breast cancer	127	0.393034147	1.686610339	0.001733	0.013287	0.004865	1987	tags=30%, list=16%, signal=25%
hsa05164	Influenza A	147	0.375373545	1.637882833	0.003407	0.013287	0.004865	2687	tags=33%, list=22%, signal=26%
hsa04145	Phagosome	131	0.374437407	1.615484317	0.001718	0.013287	0.004865	2042	tags=27%, list=17%, signal=22%
hsa04062	Chemokine signaling pathway	150	0.366717936	1.600201318	0.00346	0.013287	0.004865	2953	tags=38%, list=24%, signal=29%
hsa05166	HTLV-I infection	228	0.335745463	1.54292793	0.005128	0.01637	0.005994	1805	tags=26%, list=15%, signal=22%
hsa05418	Fluid shear stress and atherosclerosis	127	0.358494349	1.538391207	0.003466	0.013287	0.004865	1800	tags=27%, list=15%, signal=23%
hsa04015	Rap1 signaling pathway	185	0.339457452	1.52564328	0.005236	0.01637	0.005994	3098	tags=39%, list=26%, signal=30%
hsa04151	PI3K-Akt signaling pathway	296	0.318663256	1.50687448	0.003289	0.013287	0.004865	2717	tags=32%, list=23%, signal=25%
hsa04024	cAMP signaling	164	0.335610998	1.478233595	0.005338	0.01637	0.005994	1693	tags=23%, list=14%, signal=20%
hsa05200	Pathways in cancer	359	0.306356067	1.474793486	0.001631	0.013287	0.004865	2781	tags=33%, list=23%, signal=26%
hsa05169	Epstein-Barr virus infection	180	0.328066429	1.470768329	0.006897	0.019828	0.00726	1820	tags=24%, list=15%, signal=21%
hsa04072	Phospholipase D signaling pathway	127	0.337684834	1.449092242	0.010399	0.024084	0.008818	2594	tags=31%, list=22%, signal=25%
hsa05202	Transcriptional misregulation in cancer	158	0.327077602	1.439507291	0.010471	0.024084	0.008818	2922	tags=39%, list=24%, signal=30%
hsa05205	Proteoglycans in	181	0.321022037	1.439079345	0.008636	0.022069	0.00808	3116	tags=37%, list=26%, signal=28%
hsa05152	Tuberculosis	156	0.326144661	1.437781314	0.013675	0.028594	0.010469	2636	tags=29%, list=22%, signal=23%
hsa04921	Oxytocin signaling pathway	124	0.332607729	1.412995345	0.017575	0.035078	0.012843	3146	tags=32%, list=26%, signal=24%
hsa04310	Wnt signaling pathway	120	0.332640542	1.411004071	0.021053	0.035867	0.013132	3449	tags=42%, list=29%, signal=31%
hsa04360	Axon guidance	142	0.321238373	1.395461995	0.018836	0.035078	0.012843	1980	tags=25%, list=16%, signal=21%
hsa04010	MAPK signaling	228	0.301017983	1.383336801	0.008547	0.022069	0.00808	2780	tags=29%, list=23%, signal=22%
hsa04014	Ras signaling pathway	194	0.299335421	1.352703041	0.019064	0.035078	0.012843	2953	tags=33%, list=24%, signal=25%

hsa04630	Jak-STAT signaling pathway	131	0.311379313	1.34342453	0.02921	0.047987	0.01757	2682	tags=28%, list=22%, signal=22%
hsa04810	Regulation of actin cytoskeleton	185	0.297409971	1.336666852	0.020942	0.035867	0.013132	3569	tags=39%, list=30%, signal=28%
hsa04080	Neuroactive ligand-receptor interaction	233	-0.28779662	-1.389591328	0.011933	0.026139	0.00957	2195	tags=27%, list=18%, signal=22%
hsa03013	RNA transport	127	-0.35217684	-1.576073147	0.002353	0.013287	0.004865	2418	tags=31%, list=20%, signal=25%
hsa05016	Huntington's disease	153	-0.39189655	-1.779899192	0.002427	0.013287	0.004865	3343	tags=43%, list=28%, signal=32%
